# MAP Kinase FgHog1 and Importin β FgNmd5 Regulate Calcium Homeostasis in *Fusarium graminearum*

**DOI:** 10.3390/jof9070707

**Published:** 2023-06-28

**Authors:** Lixin Zhang, Yiqing Li, Lanlan Dong, Kewei Sun, Hao Liu, Zhonghua Ma, Leiyan Yan, Yanni Yin

**Affiliations:** 1 Key Laboratory of Biology of Crop Pathogens and Insects of Zhejiang Province, Institute of Biotechnology, Zhejiang University, 866 Yuhangtang Road, Hangzhou 310058, China; feiliuzhao@163.com (L.Z.); yiqingli@zju.edu.cn (Y.L.); 12116085@zju.edu.cn (L.D.); 21816169@zju.edu.cn (K.S.); 19825008848@163.com (H.L.); zhma@zju.edu.cn (Z.M.); 2 Ningbo Academy of Agricultural Sciences, Ningbo 315040, China

**Keywords:** calcium homeostasis, the HOG pathway, importin β

## Abstract

Maintaining cellular calcium (Ca^2+^) homeostasis is essential for many aspects of cellular life. The high-osmolarity glycerol (HOG) mitogen-activated protein kinase (MAPK) pathway responsible for signal integration and transduction plays crucial roles in environmental adaptation, especially in the response to osmotic stress. Hog1 is activated by transient Ca^2+^ increase in yeast, but the functions of the HOG pathway in Ca^2+^ homeostasis are largely unknown. We found that the HOG pathway was involved in the regulation of Ca^2+^ homeostasis in *Fusarium graminearum*, a devastating fungal pathogen of cereal crops. The deletion mutants of HOG pathway displayed increased sensitivity to Ca^2+^ and FK506, and elevated intracellular Ca^2+^ content. Ca^2+^ treatment induced the phosphorylation of FgHog1, and the phosphorylated FgHog1 was transported into the nucleus by importin β FgNmd5. Moreover, the increased phosphorylation and nuclear accumulation of FgHog1 upon Ca^2+^ treatment is independent of the calcineurin pathway that is conserved and downstream of the Ca^2+^ signal. Taken together, this study reported the novel function of FgHog1 in the regulation of Ca^2+^ homeostasis in *F. graminearum*, which advance the understanding of the HOG pathway and the association between the HOG and calcineurin pathways in fungi.

## 1. Introduction

In eukaryotic cells, mitogen-activated protein kinase (MAPK) pathways are responsible for numerous signal integrations, and transducing extracellular signals into the nucleus in response to a wide range of environmental stresses. Among them, the high-osmolarity glycerol (HOG) pathway has been widely studied to play a key role in cell response to hyperosmotic stress [1]. In *Saccharomyces cerevisiae*, Hog1 MAPK, a central component of the HOG pathway, is rapidly activated by two branches of upstream osmosensors that converge at Pbs2 MAPK kinase [2]. Phosphorylation of Hog1 by the MAPK cascade results in its transient accumulation in the nucleus to induce the expression of a large number of osmostress-responsive genes [3]. In addition to osmotic stress, Hog1 is also involved in the counteraction of other cellular stresses, such as oxidative stress [4,5], DNA damage [6], cadmium [7,8], temperature changes [9,10], fludioxonil [11] and tebuconazole [12]. Hyperosmotic stress induces a transient increase in the cytoplasmic calcium (Ca^2+^) concentration in eukaryotic cells [13,14,15,16], suggesting an association between the HOG and Ca^2+^ signaling pathways. In *S. cerevisiae*, Hog1 is immediately activated and accumulated in the nucleus upon transient Ca^2+^ elevation, and required for the Ca^2+^-induced expression of the glyoxalase 1 encoding gene *GLO1* through Msn2/4 transcription factors [17]. Additionally, the calcineurin pathway downstream of the Ca^2+^ signal was reported to dysregulate the HOG pathway in *S. cerevisiae* [17,18]. However, the response of the HOG pathway to rapid cytoplasmic Ca^2+^ accumulation and the cross-talk between the HOG and calcineurin pathways are largely unknown in fungi.

The nucleus translocation of phosphorylated Hog1 is required for its function in stimulating stress-induced gene expression. In general, the nucleoplasmic transport of biomacromolecules (more than 30 kDa) is mediated by karyopherins (importin/exportin/transportin), which are a superfamily of transport receptors through the nuclear pore complex (NPC) [19]. The nuclear localization signals (NLSs) on cargos are recognized by importins to form importin–cargo complexes [20]. Ran, a Ras-related small GTPase determines the direction of cargo transport [21]. In the cytoplasm, Ran binds to GDP to form RanGDP, which further binds to the importins-cargo complex that is capable of traversing NPC. In the nucleus, RanGTP replaces RanGDP to bind to the importin–cargo complex and facilitates the dissociation of importin from its cargo. Exportins bind to cargos containing nuclear export signals (NESs), then combine with RanGTP to form a trimeric complex, which passes through the NPC. In cytoplasm, RanGTP is hydrolyzed by RanGAP leading to dissociation of cargo [22,23]. In *S. cerevisiae*, importin β Nmd5 is responsible for nuclear translocation of phosphorylated Hog1 under osmotic stress, and exportin XPO1 mediates the export of dephosphorylated Hog1 from nucleus to cytoplasm [24]. Tebuconazole treatment can lead to the accumulation of activated Hog1 in nucleus in *F. graminearum*, while Ca^2+^ transient elevation and the late phase of the endoplasmic reticulum stress response all can lead to the accumulation of activated Hog1 in the nucleus of *S. cerevisiae* [12,17,25]. However, the importins which are required for the translocation of Hog1 into the nucleus upon different stresses have not been identified.

The plant pathogen *F. graminearum* causes Fusarium head blight (FHB) on triticeae crops and other important cereal crops, which is devastating to cereal crops worldwide [26]. In addition to economic losses such as yield reduction and quality decline, FHB infection also leads to the production of mycotoxins such as deoxynivalenol (DON) and zearalenone in cereals, which seriously threatens human and animal health [27,28]. In this study, we found that the mutants of the HOG pathway displayed increased sensitivity to Ca^2+^ and the calcineurin inhibitor FK506 in *F. graminearum*. Further, we explored the molecular mechanism of Ca^2+^ homeostasis regulation mediated by FgHog1, which provides a novel insight into the functions of the HOG pathway and the association between the HOG and calcineurin pathways in fungi.

## 2. Materials and Methods

### 2.1. Fungal Strains and Culture Conditions

*Fusarium graminearum* wild-type strain PH-1 (NRRL 31084) was used as a parental strain for constructing gene deletion mutants in this study. PH-1 and the resulting transformants were cultured at 25 °C on minimal medium (MM, 2 g NaNO_3_, 0.5 g KCl, 1 g KH_2_PO_4_, 0.01 g FeSO_4_, 0.5 g MgSO_4_, 30 g Sucrose, 200 μL trace elements and 1 L water, pH 6.9) for mycelia growth analysis. Fresh mycelia used for protein extraction were inoculated in yeast extract peptone dextrose (YEPD, 1%yeast extract, 2% peptone, 2% glucose) liquid medium at 25 °C for 12 h in a shaker (180 rpm). The experiment was repeated three times.

To determine sensitivity phenotypes, 5 mm (diameter) mycelial plugs of each strain taken from the edge of a 3-day-old colony were inoculated on MM or complete medium (CM, 50 mL 20 × nitrate salts, 1 g casamino acid, 10 g D-glucose, 2 g peptone, 1 g yeast extract, 1 mL trace elements, 1 mL vitamin solution and 1 L water, pH 6.5) supplemented with or without CaCl_2_ or calcineurin inhibitor FK506, and then incubated at 25 °C for 3 days. Three biological replicates were used for each strain and each experiment was repeated three times independently.

### 2.2. Construction of Gene Deletion Mutants and Complemented Strains

The double-joint PCR approach was used to generate the gene replacement construct for each target gene [29]. In brief, the up-stream and down-stream flanking regions of each gene were amplified using the primers listed in Table S1 and the flanking sequences were fused with the hygromycin-resistance gene cassette (*HPH*) driven by the constitutive trpC promoter, which was amplified from the pBS-HPH1 vector [30]. The fusion fragments were transformed using the polyethyleneglycol (PEG)-mediated *F. graminearum* protoplast transformation based on the previous study [31]. Mycelial plugs were transferred into a 250 mL flask containing 100 mL of liquid YEPD and incubated for 18 h at 175 rpm and 25 °C. After being isolated by a sterile filter, 0.2 g of fresh mycelia was incubated with 10 mL lysis buffer (0.7 M NaCl, 0. 25% driselase, 2% lysozyme, 2% cellulose) for 4 h at 30 °C. Then, protoplasts were collected via filtrate and washed twice with STC buffer (0.8 M sorbitol, 0.05 M Tris, pH 8.0, 50 mM CaCl_2_). The protoplasts were resuspended in 800 μL STC buffer and added to 35 μg construct, 20 μL spermidine and 200 μL SPTC for transformation. After 45 min, 400 μL SPTC was added into the suspension and incubated at 25 °C for 20 min. Protoplasts were transferred into 20 mL RM liquid medium (0.5 g yeast extract, 0.5 g casein hydrolysate 0.7 M sucrose and 1 L water) and incubated at 25 °C for 16 h, and then RM liquid medium containing protoplasts was mixed into PDA containing 100 μg/mL hygromycin B, and poured into plates. After incubation at 25 °C for 4 days, the importin β deletion mutants ΔFgNmd5, ΔFgKap123, ΔFgPse1, ΔFgKap114, ΔFgKap104 and ΔFgKap95 were screened by PCR assays with relevant primers (Supplementary Table S1), and used for studying the nuclear import of FgHog1. The HOG pathway deletion mutants ΔFgSsk2 (MAPK kinase kinase), ΔFgPbs2 (MAPK kinase) and ΔFgHog1 (MAPK) were obtained from our previous studies [12], and used for studying the response of the HOG pathway to rapid cytoplasmic Ca^2+^ accumulation.

To construct the FgHog1-GFP (green fluorescent protein) cassette, the double-joint PCR approach was used to amplify FgHog1 containing the promoter region and open-reading frame (without the stop codon). The resulting PCR products were co-transformed with XhoI-digested pYF11 into the yeast strain XK1-25 using the Alkali-CationTM Yeast Transformation Kit (MP Biomedicals, Solon, USA) to generate the recombined FgHog1-GFP fusion vector [32]. Subsequently, the FgHog1-GFP fusion vector was recovered from the yeast transformant using the Yeast Plasmid Kit (Solarbio, Beijing, China) and then transferred into Escherichia coli strain DH5α (TransGen Biotech, Beijing, China) for amplification. Using the similar strategy, FgSsk2-GFP, FgPbs2-GFP, FgNmd5-GFP and FgSfl1-GFP fusion cassettes were constructed. Each fusion cassette was transformed into the corresponding mutant using PEG mediated protoplast transformation.

### 2.3. Ca^2+^ Signals Measurement

For intracellular Ca^2+^ imaging, mycelia of each strain were inoculated into YEPD for 12 h at 25 °C in a shaker with 180 rpm, and then were harvested after washing 3 times with PBS. A green fluorescent Ca^2+^ binding dye Fluo-8 AM (ab142773, Abcam, Cambridge, MA, USA) was used to detect the intracellular Ca^2+^. After PBS washing, mycelia were incubated with Fluo-8 AM at the concentration of 2 μM in PBS for 30 min at 37 °C and then washed with PBS three times. Subsequently, the treated mycelia were incubated with PBS without Fluo-8 AM for 20 min at 37 °C to ensure adequate conversion of Fluo-8 AM to Fluo-8. Fluorescent signals were excited at a wavelength of 488 nm using a Zeiss LSM780 confocal microscope (Gottingen, Niedersachsen, Germany). Fluorescence intensity was quantified using ImageJ software (v1.8.0, National Institutes of Health). The experiment was repeated three times.

For quantitative assessment of Ca^2+^ concentration, fresh mycelia of each strain were inoculated in 20 mL of CMC (1 g NH_4_NO_3_, 1 g KH_2_PO_4_, 0.5 g MgSO_4_, 1 g yeast extract, 15 g sodium carboxymethylcellulose and 1 L water) liquid medium at 25 °C for 4 days in a shaker with 180 rpm to obtain conidia. The number of conidia in each flask was determined using a hemacytometer (Qiujing, Shanghai, China). A total of 10^5^ conidia of each strain was loaded with Fluo-8 AM and the procedures were performed as described above and then plated in clear-bottomed 96-well plates, respectively. BioTek Cytation 5 cell imaging multi-mode readers were used to evaluate Fluo-8 fluorescence intensity. The experiment was repeated three times with similar results.

### 2.4. Western Blotting Assay

Mycelial plugs of each strain were incubated in 150 mL YEPD liquid medium for 36 h at 25 °C with 180 rpm. Mycelia were harvested after washing 3 times with ddH_2_O. Protein samples were extracted as described previously [33]. After being finely ground in liquid nitrogen, mycelia powder was resuspended in 1 mL of protein isolation buffer (50 mM Tris-HCl, pH7.5, 100 mM NaCl, 5 mM EDTA, 1% Triton X-100, 2 mM phenylmethylsulfonyl fluoride) containing 1% protease inhibitor (Sangon Biotech, Shanghai, China). After mixture with a vortex shaker, the homogenate was incubated for 20 min on ice. Then, the homogenate was centrifuged for 15 min at 4 °C at 12,000× *g* to obtain protein samples. The protein samples separated on the 12% sodium dodecyl sulfate (SDS)-polyacrylamide gels were transferred onto Immobilon-P transfer membrane (Millipore, Billerica, MA, USA) with a Bio-Rad electroblotting apparatus. Total protein of FgHog1 and the phosphorylated FgHog1 were detected using the anti-Hog1 antibody (sc-165978, Santa Cruz, CA, USA; 1:1000 dilution) and anti-phospho-p38 antibody (#9211, Cell Signaling Technology, Boston, MA, USA; 1:1000 dilution). The monoclonal anti-GFP (ab32146, Abcam, Cambridge, UK, 1:10,000 dilution) was used to detect GFP-tagged proteins. The samples were also detected with the monoclonal anti-GAPDH antibody (EM1101, HuaAn Biotechnology, Hangzhou, China, 1:5000 dilution) or monoclonal anti-H3 antibody (ab8895, Abcam, Cambridge, UK, 1:10,000 dilution) as a reference. The experiment was conducted three times independently.

### 2.5. Nuclear–Cytoplasmic Fractionation

The wild-type PH-1, ΔFgNmd5 and ΔFgSsk2 strains were incubated and harvested for nuclear–cytoplasmic fractionation experiment as described previously [34]. About 2 g fresh mycelia of each strain were ground into fine powder in liquid nitrogen, and gently suspended with 4 mL lysis buffer (20 mM Tris-HCl pH 7.5, 20 mM KCl, 2 mM EDTA, 2.5 mM MgCl_2_, 25% glycerol, 250 mM sucrose and 5 mM DTT) containing 1:100 *v/v* protease inhibitor cocktail (C600382, Sangon Biotech, Shanghai, China) for 15 min at 4 °C. The homogenate was filtered through cell strainers (40 μm, F613461, Sangon Biotech, Shanghai, China) and then centrifuged at 1500× *g* for 10 min at 4 °C. The supernatant consisting of the cytoplasmic fraction was centrifuged at 10,000× *g* for 15 min at 4 °C and collected. The precipitate consisting of the nuclear fraction was gently washed five times with 5 mL of nuclear resuspension buffer (NRBT, 20 mM Tris-HCl pH 7.5, 2.5 mM MgCl_2_, 25% glycerol, 0.2% Triton X-100) and centrifuged at 1500× *g* for 10 min at 4 °C. The final nuclear pellet was resuspended in 800 μL lysis buffer. Protein samples were analyzed with Western blotting assay with the anti-Hog1 antibody. As quality controls for the fractionation, GAPDH was detected by using monoclonal anti-GAPDH antibody as cytoplasmic markers and histone H3 was detected by using monoclonal anti-H3 antibody as nuclear markers.

### 2.6. Yeast Two-Hybrid (Y2H) Assays

To construct plasmids for Y2H analyses, the coding sequence of each tested gene was amplified from cDNA of the wild type, with primer pairs indicated in Table S1. The cDNA fragment of each gene was inserted into the yeast GAL4-binding domain vector pGBKT7 (Clontech, Mountain View, CA, USA) and GAL4 activation domain vector pGADT7 (Clontech, Mountain View, CA, USA), respectively. The pairs of Y2H plasmids were co-transformed into *S. cerevisiae* strain Y2H Gold following the lithium acetate/single-stranded DNA/polyethylene glycol transformation protocol. Next, 2 mL Y2H Gold liquid was centrifuged for 1 min at 12,000× g and washed twice with ddH_2_O. The precipitate was resuspended in 1 mL 0.1 M LiAc and incubated for 5 min at 30 °C. Subsequently, pairs of Y2H plasmids were added into the precipitate and incubated for 20 min at 42 °C. In addition, a pair of plasmids, pGBKT7-53 and pGADT7-T, served as a positive control, and another pair of plasmids, pGBKT7-Lam and pGADT7-T, served as a negative control. Transformants were grown at 30 °C for 3 days on synthetic medium (SD) lacking Leu and Trp, and subsequently transferred to SD stripped of Leu, Trp, His and Ade (SD-Leu-Trp-His-Ade) to assess binding activity. The experiment was conducted three times independently.

### 2.7. Co-Immunoprecipitation (Co-IP) Assay

For Co-IP assays, the extracted total proteins were incubated with the GFP-trap agarose (ChromoTek, Martinsried, Germany) for 4-6 h at 4 °C with rotation. After being washed six times with 1 × TBS buffer (0.6 M Tris, 4.5 M NaCl, pH 7.4) at 4 °C, proteins binding to GFP-trap agarose were eluted and analyzed with Western blotting assay with the anti-Hog1 antibody and the monoclonal anti-GFP as described in the previous section. The monoclonal anti-GAPDH antibody was also used as a reference. The experiment was conducted three times independently.

### 2.8. Bimolecular Fluorescence Complementation (BiFC) Assay

For the BiFC assay, the PHZ65 vector carrying NYFP (N-terminal yellow fluorescent protein) and PHZ68 vector carrying CYFP (C-terminal yellow fluorescent protein) were used for generating the protein expression construct. The full lengths of FgNmd5 and FgHog1 were amplified and co-transformed with XhoI-digested PHZ65 into the yeast strain XK1-25 using the Alkali-CationTM Yeast Transformation Kit. The full length of FgHog1 was amplified and co-transformed with XhoI-digested PHZ68 into the yeast strain XK1-25. Then, the FgNmd5-NYFP and FgHog1-CYFP fusion constructs were recovered from the yeast-positive transformant using the Yeast Plasmid Kit and transferred into Escherichia coli strain DH5α for amplification. Subsequently, construct pairs of FgNmd5-NYFP and FgHog1-CYFP were co-transformed into PH-1::H1-RFP (red fluorescent protein) strain (a strain that expressed RFP-tagged histone 1 protein in PH-1 to visualize nuclei), respectively. As negative controls, construct pairs of FgNmd5-NYFP and CYFP, and NYFP and FgHog1-CYFP, were co-transformed into PH-1::H1-RFP strain. Transformants resistant to both hygromycin and neomycin were isolated and confirmed with PCR. YFP signals were examined with a Zeiss LSM780 confocal microscope. The experiment was repeated three times.

### 2.9. Conidiation, Virulence and DON Production

For conidiation assays, fresh mycelia of each strain were inoculated in a 50 mL flask containing 20 mL of CMC liquid medium. The flasks were incubated at 25 °C for 4 days in a shaker running at 180 rpm. The number of conidia in each flask was determined using a hemocytometer. In addition, septum was examined after conidia of each strain were stained with calcofluor white (CFW). Conidial morphology was observed with a Leica TCS SP5 imaging system. Three biological replicates were used for each strain and each experiment was repeated three times independently.

To assess virulence on flowering wheat heads, 10 μL spore suspension (10^5^ mL^−1^) of each strain was injected into one spikelet in flowering wheat heads of the FHB susceptible variety Jimai 22, while the control treatments were inoculated with 10 μL of sterile ddH_2_O. There were 20 replicates for each strain. After 15 days post inoculation, the infected spikelets in each inoculated wheat head were recorded. The experiment was repeated three times, and typical symptoms were shown. Wheat (*Triticum aestivum*) cultivar Jimai22 was used for coleoptile infection. Three days after sowing, the top 2 mm to 3 mm of the coleoptiles was removed. Then, 5 mm (diameter) mycelial plugs of each strain taken from the edge of a 3-day-old colony were inoculated on the top of wounded coleoptiles. After inoculation, the seedlings were grown in a growth chamber at 25 °C and 95% humidity for 3 days before examination for the infection. The experiment was repeated three times.

To determine the DON production, each strain was grown in TBI liquid medium at 28 °C for 7 days in a shaker (150 rpm) in the dark. DON Quantification Kit Wis008 (Wise Science, Zhenjiang, China) was used to quantify the DON production for each sample. The experiment was repeated three times.

## 3. Results

### 3.1. MAPK FgHog1 Is Phosphorylated upon Ca^2+^ Treatment

**Figure 1:**
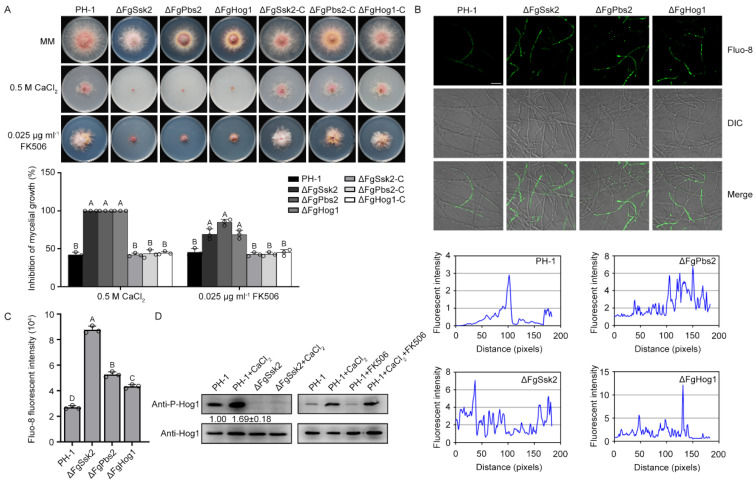
The HOG signaling pathway participates in the regulation of calcium (Ca^2+^) homeostasis in *Fusarium graminearum*. (**A**) Growth phenotype of the wild-type strain PH-1, deletion mutants of the HOG cascade kinases and the complemented strains on minimal medium (MM) with or without 0.5 M CaCl_2_ or 0.025 μg ml^−1^ calcineurin inhibitor FK506 after 3 days of incubation at 25 °C. Mycelial growth inhibition of each strain under each treatment was quantified. Line bars indicate standard deviations of three repeated experiments. The same letter on the bars indicates no significant difference according to the LSD test at *p* = 0.05. (**B**) The level of free intracellular Ca^2+^ of PH-1, ΔFgSsk2, ΔFgPbs2 and ΔFgHog1 was determined by a laser scanning microscope with 2 μM green fluorescent Ca^2+^ binding dye Fluo-8 AM after each strain was cultured in yeast extract peptone dextrose (YEPD) for 12 h at 25 °C. Fluorescence intensity was quantified using ImageJ software. Bar = 20 μm. (**C**) Total 10^5^ conidia of PH-1, ΔFgSsk2, ΔFgPbs2 and ΔFgHog1 were prepared by incubating in carboxymethyl cellulose (CMC) for 4 days at 25 °C for quantitative assessment of total Ca^2+^ content. Fluo-8 fluorescence intensity of each strain was quantified by using BioTek Cytation 5 cell imaging multi-mode readers. Line bars indicate standard deviations of three repeated experiments. The same letter on the bars indicates no significant difference according to the LSD test at *p* = 0.05. (**D**) The phosphorylation of FgHog1 was increased by the treatment with 0.15 M CaCl_2_ for 5 min and was not detected in ΔFgSsk2. Moreover, the increased phosphorylation of FgHog1 was independent of FK506. The relative intensities of phosphorylated FgHog1 bands were quantified with ImageJ software.

MAPK Hog1, a major component of the high-osmolarity glycerol mitogen-activated protein kinase (MAPK/HOG) signaling pathway, is mainly associated with regulation of cellular responses to hypertonic stress in *S. cerevisiae*. The external hypertonic signal is transduced to MAPK kinase kinases Ssk2 and Ssk22, which further activates the MAPK kinase Pbs2 and phosphorylates Hog1 by Pbs2 [1,6]. To investigate whether the HOG signaling pathway is involved in Ca^2+^ homeostasis in *Fg*, we determined the sensitivity of the pathway mutants to Ca^2+^ and calcineurin inhibitor FK506. The MAPK kinase kinase FgSsk2, MAPK kinase FgPbs2 and MAPK FgHog1 deletion mutants were obtained in our previous study [12]. As shown in Figure 1A, ΔFgSsk2, ΔFgPbs2 and ΔFgHog1 exhibited dramatically increased sensitivity to Ca^2+^ and FK506. The levels of free intracellular Ca^2+^ in these three mutants were higher than the wild-type strain PH-1 using green fluorescent Ca^2+^ binding dye Fluo-8 AM (Figure 1B,C). To further confirm that the increased sensitivity of the HOG pathway mutants to Ca^2+^ and FK506 is due to the deletion of the genes, the mutants were complemented with full-length wild-type *FgSSK2*, *FgPBS2* and *FgHOG1* genes amplified with the primers listed in Table S1, respectively. The complemented strains, ΔFgSsk2-C, ΔFgPbs2-C and ΔFgHog1-C, restored the sensitivity to Ca^2+^ and FK506 (Figure 1A). Further, we tested the phosphorylation level of FgHog1 under the treatment with Ca^2+^, and found that the phosphorylation level of FgHog1 was increased by Ca^2+^ treatment, and was abolished in ΔFgSsk2 with or without Ca^2+^ treatment (Figure 1D). In addition, FK506 treatment did not affect the Ca^2+^-induced phosphorylation of FgHog1. These data suggest that the HOG signaling pathway is involved in the regulation of Ca^2+^ homeostasis, which is independent on calcineurin pathway in *F. graminearum*.

### 3.2. Phosphorylated FgHog1 Is Transported into the Nucleus under Ca^2+^ Treatment

**Figure 2:**
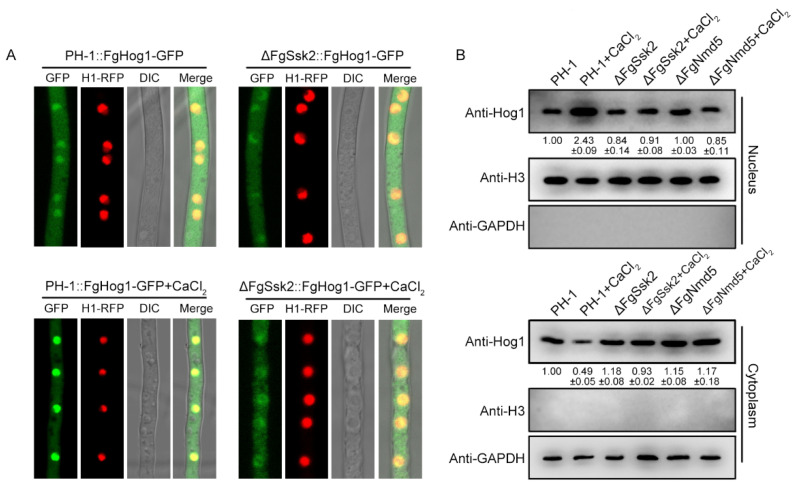
Nuclear translocation of FgHog1 relies on its phosphorylation. (**A**) Subcellular localization of FgHog1-GFP in the wild-type strain PH-1 and ΔFgSsk2 with or without 0.15 M CaCl_2_ for 5 min. Nuclei were visualized with RFP-labeled histone H1. Bar = 5 μm. (**B**) Western blots of FgHog1 in nuclear and cytoplasmic fractions of PH-1, ΔFgSsk2 and ΔFgNmd5 with or without 0.15 M CaCl_2_ for 5 min, respectively. Histone H3 and GAPDH were used as markers of the nuclear and cytoplasmic fractions. The relative intensities of nuclear or cytoplasmic fractions of FgHog1 bands were quantified with ImageJ.

In phytopathogenic fungi, phosphorylated Hog1 was transported into the nucleus under osmotic stress but not under oxidative stress [2]. We therefore were interested in whether phosphorylated FgHog1 is transported into the nucleus upon Ca^2+^ treatment. The FgHog1-GFP (green fluorescent protein) construct was generated and transformed into PH-1 and the ΔFgSsk2 mutant strain. Confocal microscopic examination showed that FgHog1-GFP entered into the nucleus in PH-1, but not in ΔFgSsk2 upon Ca^2+^ treatment (Figure 2A), suggesting that the phosphorylation is required for its nuclear import. Nuclear/cytoplasmic fractionation assays further confirmed the accumulation of FgHog1 in the nucleus in PH-1 upon Ca^2+^ treatment (Figure 2B).

### 3.3. Identification of Nuclear Importin α and β in F. graminearum

**Figure 3:**
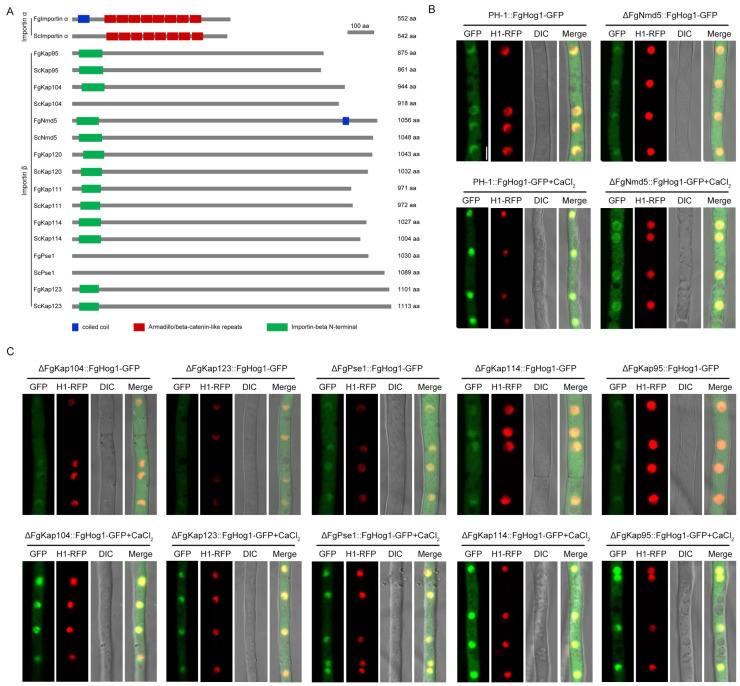
Identification of nuclear importin α and β in *F. graminearum*. (**A**) Domain structures of putative importin family proteins from *Fusarium graminearum* and *Saccharomyces cerevisiae* were analyzed with the SMART protein database (http://smart.embl-heidelberg.de (accessed on 21 August 2022)) and the NBCI protein database (https://blast.ncbi.nlm.nih.gov/Blast.cgi (accessed on 21 August 2022)). (**B**,**C**) Subcellular localization of FgHog1-GFP with or without 0.15 M CaCl_2_ for 5 min in the deletion mutants of the wild-type strain PH-1 and six importin βs. Bar = 5 μm.

The classical nuclear import pathway relies on importin α and β: importin α as an adaptor protein links cargo protein to importin β; then importin β translocates the importin α/importin β/cargo protein complex to the cell nucleus by transiently interacting with the NPC [35]. To explore the mechanism of FgHog1 entering the nucleus, a total of one putative importin α and eight putative importin βs (FgKap95, FgKap104, FgNmd5, FgKap120, FgKap111, FgKap114, FgPse1 and FgKap123) were identified by BLASTp searching the *F. graminearum* genome database (http://fungi.ensembl.org/Fusarium_graminearum/Info/Index (accessed on 21 August 2022)) with importin homologs from *S. cerevisiae* as queries (Figure 3A). Then, we knocked out eight importin βs from the strain PH-1::FgHog1-GFP using the homologous recombination strategy, each gene were screened by PCR with the primer pairs as listed in Table S1. We obtained six importin β deletion mutants ΔFgKap104::FgHog1-GFP, ΔFgKap123::FgHog1-GFP, ΔFgPse1::FgHog1-GFP, ΔFgNmd5::FgHog1-GFP, ΔFgKap114::FgHog1-GFP and ΔFgKap95::FgHog1-GFP (Supplementary Figure S1), since we were unable to delete FgKap111 and FgKap120 after obtaining more than 100 ectopic transformants from three independent transformation experiments.

Subcellular localization observation showed that FgKap104, FgKap123, FgPse1, FgKap114 and FgKap95 had no effect on FgHog1 translocation with or without Ca^2+^ treatment, while the lack of FgNmd5 impeded the nuclear accumulation of FgHog1 localization upon Ca^2+^ treatment (Figure 3B,C).

### 3.4. The Importin β FgNmd5 Is Responsible for Transporting the Phosphorylated FgHog1 into Nuclear

**Figure 4:**
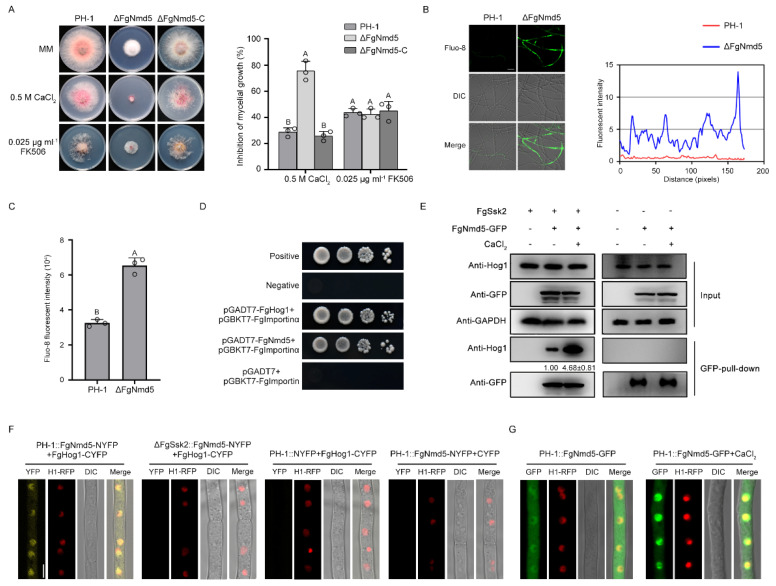
The importin β FgNmd5 is responsible for transporting the phosphorylated FgHog1 into the nucleus. (**A**) Sensitivity of PH-1 and ΔFgNmd5 to 0.5 M CaCl_2_ and 0.025 μg mL^−1^ FK506. A 5 mm mycelial plug of each strain was inoculated on MM with or without CaCl_2_ and FK506 and incubated for 3 days at 25 °C. Mycelial growth inhibition of each strain under each treatment was quantified. Line bars indicate standard deviations of three repeated experiments. The same letter on the bars indicates no significant difference according to the LSD test at *p* = 0.05. (**B**) The level of free intracellular Ca^2+^ of PH-1 and ΔFgNmd5 was determined by a laser scanning microscope with 2 μM green fluorescent Ca^2+^ binding dye Fluo-8 AM after each strain was cultured in YEPD for 12 h at 25 °C. Fluorescence intensity was quantified using ImageJ software. Bar = 20 μm. (**C**) Total 10^5^ conidia of PH-1 and ΔFgNmd5 were prepared by incubating in CMC for 4 days at 25 °C for quantitative assessment of total Ca^2+^ content. Fluo-8 fluorescence intensity of each strain was quantified by using BioTek Cytation 5 cell imaging multi-mode readers. Line bars indicate standard deviations of three repeated experiments. The same letter on the bars indicates no significant difference according to the LSD test at *p* = 0.05. (**D**) Yeast two-hybrid (Y2H) assay verified the direct interaction of FgHog1 and FgNmd5 with FgImportin α, demonstrating the indirect association between FgHog1 and FgNmd5. A pair of plasmids, pGBKT7-53 and pGADT7-T, was used as the positive control. A pair of plasmids, pGBKT7-Lam and pGADT7-T, was used as the negative control. (**E**) Co-immunoprecipitation (Co-IP) assays verified that FgHog1 interacted with FgNmd5, and the interaction was enhanced by CaCl_2_ treatment and dependent on FgSsk2. Western blots of total proteins (input) extracted from transformants expressing the FgNmd5-GFP constructs and proteins eluted from anti-GFP agarose beads were detected with the anti-Hog1 antibody. The monoclonal anti-GAPDH antibody was used as a reference. Band intensities were quantified with ImageJ. (**F**) The interaction of FgHog1 and FgNmd5 in the nucleus and cytoplasm is dependent on FgSsk2 in bimolecular fluorescence complementation (BiFC) assays. Construct pairs of NYFP+FgHog1-CYFP and FgNmd5-NYFP+CYFP served as negative controls. YFP signals were observed using confocal microscopy. Bar = 5 μm. (**G**) Subcellular localization of FgNmd5-GFP in the wild-type strain PH-1 with or without 0.15 M CaCl_2_ for 5 min. Nuclei were visualized with RFP-labeled histone H1. Bar = 5 μm.

To identify the roles of FgNmd5 in calcium homeostasis, we generated a deletion mutant and examined its sensitivity to Ca^2+^ and FK506. As shown in Figure 4A–C, ΔFgNmd5 displayed increased sensitivity to Ca^2+^ and the elevation of intracellular Ca^2+^, but similar sensitivity to FK506 as well as the wild type, suggesting that FgNmd5 may affect the activity of calcineurin via other substrates.

Further, yeast two-hybrid (Y2H) assays showed that FgHog1 binds to FgImportin α, but not FgNmd5 (Figure 4D), indicating that the interaction of FgHog1 and FgNmd5 may occur through FgImportin α. Co-immunoprecipitation (Co-IP) and bimolecular fluorescence complementation (BiFC) assays showed that FgHog1 interacts with FgNmd5; such interaction was increased by Ca^2+^ treatment and was dependent on the phosphorylation of FgHog1 (Figure 4E,F). In addition, FgNmd5-GFP entered into the nucleus upon Ca^2+^ treatment (Figure 4G) and the nuclear/cytoplasmic fractionation assay also confirmed that phosphorylated FgHog1 is transported into the nucleus by importin β FgNmd5 (Figure 2B). These results indicate that phosphorylated FgHog1 was translocated into the nucleus via importin β FgNmd5.

### 3.5. Ca^2+^ Homeostasis Plays Important Roles in Development and Virulence

**Figure 5:**
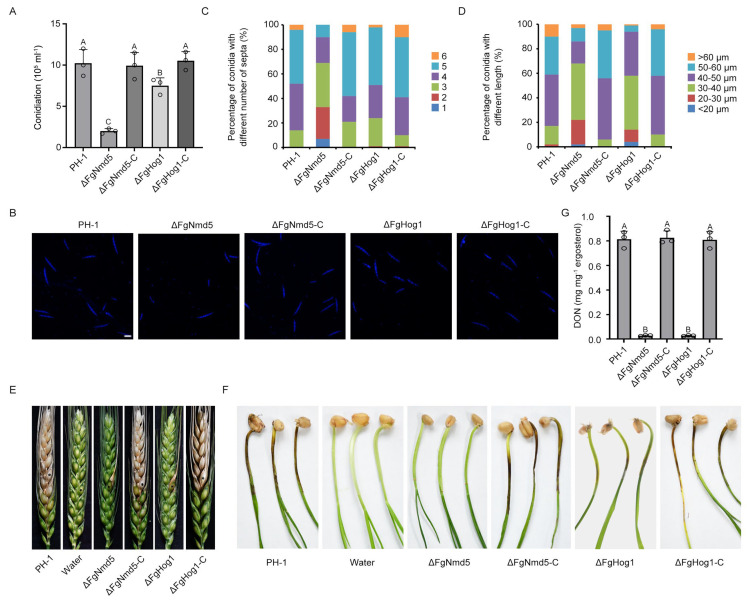
FgNmd5 and FgHog1 are important for conidiation, DON production and virulence. (**A**) Numbers of conidia produced by the wild-type PH-1, ΔFgNmd5, ΔFgHog1 and complemented strains. Line bars indicate standard deviations of three repeated experiments. The same letter on the bars indicates no significant difference according to the LSD test at *p* = 0.05. (**B**) Conidial morphology of PH-1, ΔFgNmd5, ΔFgHog1 and complemented strains. Differential interference contrast [DIC] images of conidia stained with calcofluor white (CFW) were captured with Leica TCS SP5 imaging system. Bar = 40 μm. (**C**) Percentages of conidia with different numbers of septa in PH-1, ΔFgNmd5, ΔFgHog1 and complemented strains. (**D**) Percentages of conidia with different lengths in PH-1, ΔFgNmd5, ΔFgHog1 and complemented strains. (**E**) The mutants of FgNmd5 and FgHog1 showed significantly reduced virulence on wheat heads. Infected wheat heads were determined after 15 days of inoculation with conidial suspension of PH-1, ΔFgNmd5, ΔFgHog1 and complemented strains. Sterile water was used as the control. The inoculated site on each wheat head is labeled with a black dot. (**F**) Wheat coleoptiles were inoculated with PH-1, ΔFgNmd5, ΔFgHog1 and complemented strains. Representative images were taken 5 days after inoculation. Sterile water was used as the control. (**G**) The amount of DON produced by PH-1, ΔFgNmd5 and ΔFgHog1 were determined after growth in the toxin-inducing medium (TBI) for 7 days. Values on the black bars followed by the same letter are not significantly different at *p* = 0.05.

Given that FgNmd5 and FgHog1 are all involved in calcium homeostasis, we test the phenotypes of their mutants to explore the functions of calcium homeostasis. As shown in Figure 5A, deletion of FgNmd5 and FgHog1 caused 80% and 27% reduction in conidiation, and the complemented strains ΔFgNmd5-C and ΔFgHog1-C restored the defect. Moreover, lack of FgNmd5 and FgHog1 produced abnormal conidia. Compared to wild-type PH-1 and the complemented strains, the conidia of ΔFgNmd5 were shorter with fewer septa, while the conidia of ΔFgHog1 were shorter (Figure 5B–D). The virulence of each mutant was evaluated on flowering wheat heads and coleoptiles. Lack of FgNmd5 or FgHog1 led to significantly reduced virulence (Figure 5E,F). Since DON is an important virulence factor of *F. graminearum* [36], we therefore determined DON production in the deletion mutants. As shown in Figure 5G, ΔFgNmd5 and ΔFgHog1 presented a significant decrease in DON production. Together, these results indicated that calcium homeostasis mediated by FgNmd5 and FgHog1 regulates asexual development and virulence in *F. graminearum*.

## 4. Discussion

Intracellular Ca^2+^ are important second messengers in all organisms. In fungi, the regulation of the Ca^2+^ homeostasis system maintains the cytoplasmic Ca^2+^ concentration in a range from 50 to 200 nM [37]. In general, the plasma membrane Ca^2+^ influx system is activated in response to various external stresses, and increased Ca^2+^ concentration affects a wide range of cellular processes [38,39,40]. However, a sustained elevation in cytosolic Ca^2+^ concentration is detrimental to fungal cells. The increased Ca^2+^ concentration not only modulates signaling cascades, but also activates the calcineurin pathway to reduce the Ca^2+^ concentration to the basal level [41,42]. In *S. cerevisiae*, the HOG pathway has been found to be activated by Ca^2+^ treatment [17]. In *Magnaporthe oryzae*, putative type 2C protein phosphatases MoPtc1 and MoPtc2 are induced by Ca^2+^ and also participate in the regulation of calcium homeostasis. Deletion of both MoPtc1 and MoPtc2 leads to overstimulation of Hog1 [43]. In this study, we found that the HOG pathway functions in Ca^2+^ homeostasis regulation; moreover, this role of the HOG pathway is independent of the calcineurin pathway in *F. graminearum* (Figure 1), which is a difference from studies on *S. cerevisiae* and *A. fumigates.* Shitamukai et al. showed that the calcineurin and its downstream transcription factor Crz1 antagonize the HOG pathway in growth regulation, and the sensitivity of ΔHog1 to CaCl_2_ was recovered by deleting calcineurin in *S. cerevisiae* [44]. In *A. fumigates*, the transcription factor CrzA (Crz1 ortholog) can directly bind to the promoter of *SSKB* (the MAPKKK kinase of the HOG pathway), thereby inducing the *SSKB* mRNA accumulation, and subsequently modulating SakA (Hog1 ortholog) phosphorylation upon osmotic stress [45]. In *S. cerevisiae*, methylglyoxal, a metabolite derived from glycolysis, was reported to have the ability to activate these two pathways in parallel [46]. Taken together, these results indicate that the crosstalk of the HOG and calcineurin pathways vary among different fungi under various environmental stresses.

The nucleus translocation of phosphorylated Hog1 is particularly important for the stress-response regulation. *S. cerevisiae* Hog1 has been reported to be transported into the nucleus by importin β Nmd5 upon osmotic stress [24]. However, in mammals, nuclear accumulation of p38 (Hog1 ortholog) is mediated by multiple importin βs, including Imp3, Imp7 and Imp9, under stimulus with tetradecanoyl phorbol acetate or anisomycin [47]. Our study found that phosphorylated FgHog1 is transported into the nucleus only by FgNmd5, not other importin βs under Ca^2+^ treatment (Figure 2 and Figure 3), which is in agreement with the budding yeast, and suggests that the mechanisms of importins coordinately transporting cargos are more complex in higher eukaryotes. In yeast, cells initiate transcription of osmostress-responsive genes within the first few minutes after NaCl treatment, and the nuclear accumulation of Hog1 increased at the same time. Once the cell overcome the stress, Hog1 returns to the cytoplasm and the transcriptional activation stops [48]. Although FgNmd5 has the ability to transport multiple biological macromolecules based on the studies in *S. cerevisia*, *M. oryzae* and Hela cells [49,50,51], we found that FgHog1 and FgNmd5 interact in both the nucleus and cytoplasm in a resting state in *F. graminearum* (Figure 4D–F), which provides the prerequisite for rapid response to external stimuli.

Ca^2+^ homeostasis, usually maintained by Ca^2+^ channels, exchangers, pumps and downstream signaling pathways, plays important roles in many physiological processes [52,53,54]. In *S. cerevisiae*, deletion in Golgi- and ER-associated Ca^2+^ transporters affects the repair of 4-NQO-induced DNA damage [55]. The Ca^2+^ homeostasis system in *C. albicans* is essential for virulence, stress response and drug resistance [56]. Five Vcx1 (vacuolar Ca^2+^ exchanger) paralogues in *Beauveria bassiana* are critical for different multiple stress responses and virulence [57]. One Ca^2+^/Mn^2+^-ATPase Spf1 supplies ER lumen with Ca^2+^/Mn^2+^ and regulates hyphal growth, conidiation, host penetration and pathogenicity in *M. oryzae* [58]. As Ca^2+^/Mn^2+^ transporters, FgGdt1 and FgPmr1 are essential in various development and infection processes of *F. graminearum* by maintaining the homeostasis of Ca^2+^ and manganese in the Golgi [59]. The Ca^2+^-calcineurin pathway was reported to regulate development, pathogenicity and stress responses in various fungal species [60,61]. In our study, lack of the Ca^2+^ homeostasis regulators FgHog1 or FgNmd5 led to reduced conidiation and pathogenicity (Figure 5). Taken together, these results indicate that Ca^2+^ homeostasis is critical for multiple biological processes in fungi.

## 5. Conclusions

The HOG MAPK pathway plays a crucial role in signal transduction, which is responsible for environmental adaptation in fungi. Previous reports detailed the function of the HOG pathway in overcoming osmotic stress. However, the regulatory mechanisms of the HOG pathway in calcium homeostasis are not well characterized. In this study, we found that the HOG pathway is involved in the regulation of calcium homeostasis in *F. graminearum*, a predominant causal agent of FHB. Deletion of the HOG pathway components FgSsk2, FgPbs2 or FgHog1 caused increased sensitivity to calcium and FK506 and elevated levels of free intracellular Ca^2+^. The treatment with Ca^2+^ induced the phosphorylation of FgHog1, which was subsequently translocated to the nucleus, mediated by importin β FgNmd5. Collectively, our data uncover a novel role of FgHog1 in the regulation of calcium homeostasis in *F. graminearum*.

## Data Availability

Relevant data supporting the findings of this study are available in this article and its Supplementary Information files.

## Supplementary Materials

The following supporting information can be downloaded at: https://www.mdpi.com/article/10.3390/jof9070707/s1, Figure S1: Generation and identification of the gene deletion mutants and complemented strains of *Fusarium graminearum*; Table S1: A list of PCR primers used in this study and their relevant characteristics.
