# Supplementary figures and images for: MAP Kinase FgHog1 and Importin β FgNmd5 Regulate Calcium Homeostasis in Fusarium graminearum
Source: J Fungi (Basel). 2023 Jun 28;9(7):707. doi: 10.3390/jof9070707 (PMC10381525; doi:10.3390/jof9070707)

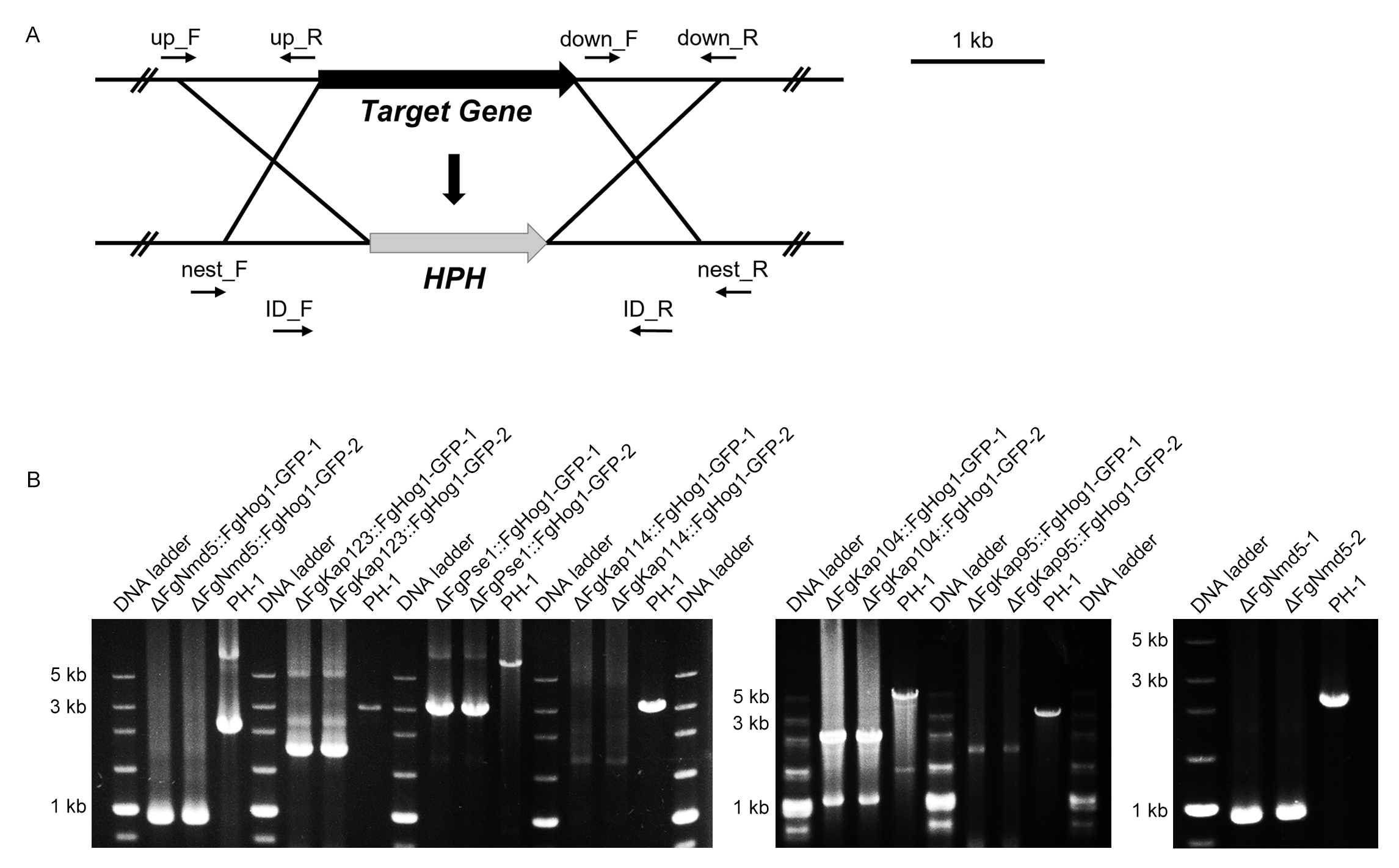

Supplement: Supplementary file 1 [file jof-09-00707-s001.zip › Figure S1.tif]
